# Identification of Allelic Imbalance with a Statistical Model for Subtle Genomic Mosaicism

**DOI:** 10.1371/journal.pcbi.1003765

**Published:** 2014-08-28

**Authors:** Rui Xia, Selina Vattathil, Paul Scheet

**Affiliations:** 1 Department of Epidemiology, The University of Texas MD Anderson Cancer Center, Houston, Texas, United States of America; 2 Division of Biostatistics, The University of Texas School of Public Health, Houston, Texas, United States of America; 3 Human & Molecular Genetics Program, The University of Texas Graduate School of Biomedical Sciences, Houston, Texas, United States of America; Thomas Jefferson University, United States of America

## Abstract

Genetic heterogeneity in a mixed sample of tumor and normal DNA can confound characterization of the tumor genome. Numerous computational methods have been proposed to detect aberrations in DNA samples from tumor and normal tissue mixtures. Most of these require tumor purities to be at least 10–15%. Here, we present a statistical model to capture information, contained in the individual's germline haplotypes, about expected patterns in the B allele frequencies from SNP microarrays while fully modeling their magnitude, the first such model for SNP microarray data. Our model consists of a pair of hidden Markov models—one for the germline and one for the tumor genome—which, conditional on the observed array data and patterns of population haplotype variation, have a dependence structure induced by the relative imbalance of an individual's inherited haplotypes. Together, these hidden Markov models offer a powerful approach for dealing with mixtures of DNA where the main component represents the germline, thus suggesting natural applications for the characterization of primary clones when stromal contamination is extremely high, and for identifying lesions in rare subclones of a tumor when tumor purity is sufficient to characterize the primary lesions. Our joint model for germline haplotypes and acquired DNA aberration is flexible, allowing a large number of chromosomal alterations, including balanced and imbalanced losses and gains, copy-neutral loss-of-heterozygosity (LOH) and tetraploidy. We found our model (which we term J-LOH) to be superior for localizing rare aberrations in a simulated 3% mixture sample. More generally, our model provides a framework for full integration of the germline and tumor genomes to deal more effectively with missing or uncertain features, and thus extract maximal information from difficult scenarios where existing methods fail.

## Author Summary

Allelic imbalance, or a deviation from the expected 1-to-1 ratio of alleles where both were present in the germline, can result when there has been an acquired deletion or duplication of part of a chromosome and is a hallmark of cancer genomes. Tumor genomic profiling studies often involve analysis of samples that contain aberrant tumor cells mixed with normal cells without these acquired mutations. Methods for detecting chromosomal aberrations that result in allelic imbalance within a heterogeneous sample have previously been proposed that use the dispersion of within-sample allele frequencies measured at germline heterozygous positions. Here we demonstrate that combining this information with a measure for the correlation in these dispersions, due to the imbalance of one of the chromosomes, provides the most powerful approach. Our method allows for sensitive identification of short allelic imbalance events (e.g. 10 Mb) contained in as few as 3% of the cells in a heterogeneous mixture. Applications include profiling tumor genomes following surgical resection where there exists high contamination of normal tissue and identifying aberrations in subclones. Our work provides a framework for further development of methods that use observed data and population genetic theory for inference of allelic imbalance.

This is a *PLOS Computational Biology* Methods article.

## Introduction

Identification of DNA copy number aberrations and loss of heterozygosity (LOH) in known or potential cancer-related genomic regions offers the potential for application in basic or translational science. Due to limits of tissue dissection, or when dissection is impractical (e.g. high vascularity or hematological cancers), a DNA sample may exhibit genetic heterogeneity resulting from the mixture of tumor and normal tissues or from subclonal structure. In such cases the task of fully characterizing the genomes present in individual tissues or clones becomes difficult.

Numerous computational methods have been proposed to detect chromosomal aberrations in DNA samples from tumor and normal tissue mixtures using single-nucleotide polymorphism (SNP) genotyping arrays. Inference of aberrations present in the DNA from heterogeneous mixtures of cells requires intermediate data features from SNP arrays, i.e. the B allele frequency (BAF, the proportion of the “B” allele in the sample) and log R ratio (LRR, indicative of total copy number), since genotype calls alone may be unaffected by the presence of a small proportion of aberrant cells. In regions of allelic imbalance (AI), the center of the distribution for a BAF of a germline heterozygous marker is shifted from the expected heterozygote BAF of 0.5, with the magnitude of the shift dependent on the event type and aberrant cell proportion, and its direction (toward either 0 or 1) dependent on the allele on the imbalanced chromosome (see [1] for examples). As a consequence, BAFs at germline heterozygous sites across an AI region form two bands that can be described with a bimodal distribution. The main strategies for accommodating this BAF pattern are use of a two-component mixture distribution and mirroring.

In one approach, the observed data are modeled as a two-component mixture. We discuss and evaluate two such methods here. genoCN [2], a discrete-state hidden Markov model (HMM) based method, uses the BAFs at germline heterozygous sites as the observed data and defines the emission function as a mixture of the distribution functions for each of the two BAF bands, with each component having equal weight at every marker. PSCN [3] uses a continuous-state HMM to model the observed allele-specific probe signal intensities, with the two components assigned equal weight. In the mirroring approach, the observed BAF values are reflected about 0.5 to create a unimodal “mirrored BAF” (mBAF). If the untransformed BAF distribution was normal, then the mirroring process reliably creates a “half-normal” distribution in non-aberrant regions. In imbalanced regions, the resulting mBAF distribution is more difficult to predict since it will depend on the magnitude of the BAF shift. If the BAF shift is large enough, then the mirroring creates a normal distribution with a shift. However, if the BAF shift is small and points in the two bands overlap the reflection point, then the newly created distribution will be distorted (bounded by and skewed toward 0.5, see examples in Supplementary Figure S1). This folding of the null distribution and distortion of the alternative distribution results in a loss of discriminatory power in cases of low levels of imbalance compared to analogous tests that use untransformed BAFs. Mirroring also interferes with the estimation of tumor proportion based on the observed data, since the function relating tumor proportion and event type is based on the normal density, as well.

Each of the aforementioned methods assume that the observed data are independent across SNP markers given the tumor DNA aberration state. However, particularly at low proportions of aberrant cells, leveraging the inherited haplotypes of the individual to define specific expected patterns of BAF shifts offers a powerful strategy to distinguish signal from noise. To illustrate the utility of this information, we present a conceptual “re-orientation” process to harness the dependence in the BAF data (Supplementary Figure S2). With complete haplotype information for a given individual, the “A/B” allele designations for each heterozygous marker could be switched so that one chromosome carried all “A” alleles at heterozygous markers while the other all “B” alleles. As a consequence, the observed BAF at a marker for which the allele label changed would be replaced with its complement (1-BAF). Importantly, although an AI-inducing aberration would still produce a “one-band” pattern after this process (as it would after mirroring), the distribution of the reoriented BAFs would maintain normality, even at the lowest levels of imbalance.

The challenge of this approach is that we do not observe the haplotypes directly from DNA arrays. Several strategies have been employed to cope with this limitation while still using haplotypes to aid in detection or interpretation of AI. The POD method [4] first uses combinations of parental genotypes to set up testing an offspring's BAF values for outliers, with BAF thresholds calibrated on aberration-free chromosome arms. Using markers with BAF outliers, POD discovers segments of abnormal representation from one parent by applying two-sided binomial tests in sliding windows. Trio-based phasing provides highly accurate haplotypes at informative loci and this method is therefore very useful in cases where trio genotypes are available. Nik-Zainal *et al* [5] statistically phased heterozygous SNP genotypes from a matched normal sample and plotted the tumor BAFs by color according to alignment with the inferred haplotypes, forming so-called Battenberg plots. A segmentation algorithm was then applied to find switch points in these plots, which were used to re-orient segments to extend haplotype estimates to whole chromosome arms and facilitate the identification of clonal and subclonal aberrations. We would expect this approach to perform well when BAFs are well diverged but fail when allelic imbalance magnitude is small. We previously suggested the use of local phase concordance between observed BAFs and statistically-estimated haplotypes to create a data transformation amenable to break-point detection algorithms for regions of AI smaller than entire chromosome arms [6]. However, this method (hapLOH) ignored the magnitude of the BAF deviations and, in its current implementation, does not utilize LRR values.

Here we propose a model for joint inference of germline haplotypes and chromosomal alterations, including balanced and imbalanced losses and gains, copy-neutral LOH, and tetraploidy (J-LOH). We use a parametric model for haplotype variation to integrate over uncertainty in haplotypes while naturally parameterizing the magnitude of AI with a combination of aberration type and proportions of the components of the mixture (tumor and normal DNA). In terms of the aberration types that are explicitly captured, our model is most similar to that underlying GPHMM [7], in which the observed data (mBAF and LRR) are related to the hidden aberration states by normal density functions. To this framework, we add a HMM for the inherited haplotypes that can be fit with existing population genetic data. Knowledge of the germline haplotypes allows us to model the expected patterns of BAFs in an AI region. Our approach does not suffer from drawbacks of some other approaches that attempt to use haplotype information in that we do not rely on sliding windows of markers but instead evaluate each marker for AI using the data at flanking SNP markers, with more proximal markers contributing more information about AI, effectively integrating over all window sizes. Our model is especially motivated for application to samples with high normal DNA contamination (>90%), where it becomes necessary to consider the correlation among BAF values, although (like other methods) it will work better at lower levels of contamination (so long as genotypes are sufficiently accurate). Below we evaluate the performance of J-LOH on a well-studied tumor cell line dilution series and a set of computationally simulated dilutions, and also present results from analysis of adjacent normal tissue from a study of the genomics of hepatocellular carcinoma.

## Results

### Lab-based dilution samples

We ran J-LOH and other methods (ASCAT [8], genoCN, GPHMM, PSCN, and hapLOH) on SNP array data from samples with various low tumor cell proportions (10%, 14%, 21% and 30%) from a well-studied paired tumor-normal cell line dilution series [1]. We applied GAP on the pure tumor cell line data to obtain a sort of “gold standard” of aberration calls for evaluating methods applied to the diluted samples. Comparisons of genome-wide call concordance between each method and the tumor-informed GAP calls are summarized in Table 1. Results are presented for 3 accuracy metrics (criteria) to accommodate output from all methods.

**Table 1:** Genome-wide concordance of aberration estimates.

	Copy number and LOH				Gain or loss				Allelic imbalance			
	30%	21%	14%	10%	30%	21%	14%	10%	30%	21%	14%	10%
J-LOH	**0.95**	0.91	**0.90**	**0.78**	**0.96**	0.92	**0.91**	**0.82**	**0.99**	**0.97**	**0.97**	**0.91**
J-LOH (*K* = 1)	0.95	**0.93**	0.86	0.73	0.96	**0.94**	0.88	0.77	0.98	0.96	0.92	0.85
GPHMM	0.94	0.90	0.67	0.51	0.94	0.92	0.68	0.66	0.97	0.96	0.82	0.82
genoCN	0.23	0.03	0.03	0.03	0.26	0.05	0.03	0.03	0.74	0.32	0.20	0.17
ASCAT	0.78	-	-	-	0.82	-	-	-	0.90	-	-	-
PSCN	n/a	n/a	n/a	n/a	0.18	0.45	0.32	0.04	n/a	n/a	n/a	n/a
hapLOH	n/a	n/a	n/a	n/a	n/a	n/a	n/a	n/a	0.97	0.95	0.89	0.82

We present aberration call concordance for different methods at different tumor purities (30%, 21%, 14%, 10%). We defined the concordance rate as the percentage of markers with calls consistent with GAP, in terms of the following: (left) Copy number and LOH status, i.e. matching both total allele copy number and existence of LOH; (middle) Gain or loss status in the aberrant cells of the two germline alleles (inherited as 1-1), i.e. “gain/gain” (2-2), “gain/normal” (3-1); or (right) Allelic imbalance, i.e. presence or absence of AI. Missing entries (“-”) indicate either no output or a small value (less than 0.01). Some cells contain “n/a” due to limits of the output from certain methods, i.e. PSCN allows comparison of gain or loss concordance only, and hapLOH simply outputs probability of allelic imbalance.

Concordance for nearly all methods decreases with tumor purity, due to decreased signal in both BAF and LRR. (PSCN actually shows a decreased concordance at 30% relative to some lower purities, as noted elsewhere [3].) Among methods considered here, J-LOH is the least adversely affected by lower tumor purity, maintaining a concordance for specifying copy number and LOH of 0.78 at the lowest purity (10%), compared with 0.51 for GPHMM. To gain a greater understanding of which innovations were producing the biggest impact in results, we implemented a simple, haplotype-free, version of our method (labeled as “*K* = 1”). Like GPHMM (and other methods except hapLOH), this version ignores haplotype information. However, we accommodate the increased dispersion in the BAFs via a mixture of two normal distributions, rather than by modeling the distorted mBAF values. Interestingly, this version of our model also appears superior to GPHMM; for example, J-LOH (*K* = 1) showed a concordance (for copy number and LOH) of 0.73, a modest decrease from the full version of J-LOH. Other methods, specifically genoCN and PSCN, had concordance values well below those obtained via J-LOH, GPHMM, and hapLOH. ASCAT failed to produce a result below 30% tumor purity, possibly due to limits embedded in its implementation.

**Figure 1:**
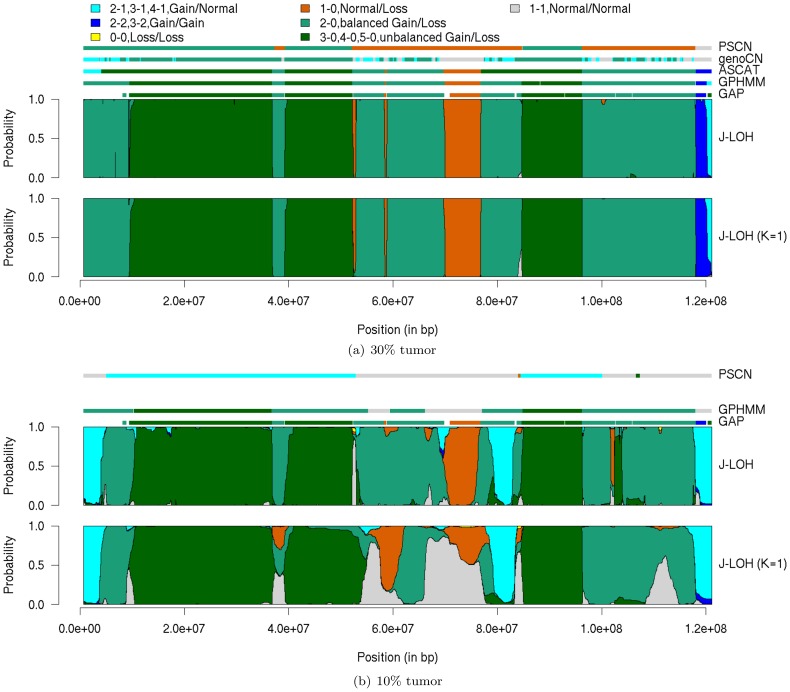
Posterior marginal probabilities for p-arm of chromosome 1. Results from J-LOH and J-LOH (*K* = 1) are presented for the 30% tumor sample (top panel) and 10% tumor sample (bottom panel). The vertical height of the colored bars at each marker is proportional to the posterior marginal probability of the corresponding aberration category. Aberration types were placed into categories based on allele copy gain or loss. Horizontal bars at the top of each panel depict the regions called by other methods, from bottom: GAP, GPHMM, ASCAT, genoCN, and PSCN. ASCAT and genoCN did not produce results in the 10% tumor sample. Empty segments of the GAP bar indicate regions with sub-clones or low confidence scores.

To investigate in more detail the results at the most difficult setting, we plotted the aberration calls for the p-arm of chromosome 1 at the 30% and 10% tumor purity dilutions. Because of the large number of aberration states, we combined certain aberration calls, such as “2-1, 3-1, 4-1, Gain/Normal” (where one chromosome is gained and the other left at copy number 1), for ease of visual evaluation of the results. This grouping scheme (enumerated completely atop the plots) is in line with the “gain or loss” metric used by PSCN. Results for PSCN, genoCN, ASCAT, GPHMM, GAP and J-LOH are displayed in Figure 1.

At the 30% purity level, all methods except PSCN and genoCN are consistent in their identification of the aberrant regions. In the 10% sample, however, there are several differences among methods. J-LOH calls the copy-neutral LOH (cn-LOH) regions at 8*e*7 bp and 1.2*e*8 bp as an unbalanced gain (possibly at the left end of the chromosome as well, though GAP's call here is not fully specified). Despite these, J-LOH appears to offer greater precision than is obtained from ignoring haplotypes, with more pronounced distinctions among states; for example, J-LOH (*K* = 1) is less confident in its call of the deletion at 7*e*7 bp than is J-LOH. GPHMM calls the “3-0” region from 4*e*7 to 5*e*7 bp as cn-LOH and misses the deletion at 7*e*7 bp. In fact, a key distinction between GPHMM and J-LOH is that GPHMM miscalls every deletion event across the genome at the 10% purity (cross-tabulation with results from GAP; data not shown). This difficulty is consistent with the lower signal expected in the mBAF quantity for the most subtle forms of AI.

### Low-purity computational dilutions

In order to test our method at even lower tumor purities, we used a computational dilution data set with targeted tumor cell proportions of 1% to 10%, simulated to mimic actual chromosomal loss or gain, thus keeping intact expected correlations among alleles (see Methods). We attempted to apply all of the methods assessed on the real dilutions; additionally, we applied hapLOH [6], which was specifically designed for low purities. However, several of the methods (PSCN, genoCN, ASCAT) did not produce output at these lower values and thus could not be scored. At these lower purities we did not attempt to score the remaining methods on their ability to differentiate among aberrant event types but rather grouped all AI events into a single aberrant class. In this most difficult setting, simply detecting the presence of an aberration becomes a more pragmatic goal. Subsequent to computational detection, laboratory-based methods may offer more informative characterization depending on the specific application. Comparisons of sensitivity among the methods are presented in Table 2.

**Table 2:** Genome-wide sensitivity and specificity for low purity simulations.

	9%	7%	5%	3%
J-LOH	**0.99(1.00)**	**0.98(0.99)**	**0.94(0.99)**	**0.64(0.99)**
J-LOH (*K* = 1)	0.97(0.99)	0.94(0.99)	0.75(0.99)	0.24(0.99)
GPHMM	0.31(1.00)	-	-	-
hapLOH	0.79(0.99)	0.52(0.99)	0.28(0.99)	0.01(1.00)
J-LOH^†^	0.98(0.99)	0.95(0.99)	0.81(0.98)	0.31(0.98)

(†) With a limited state space (normal, cn-LOH, hemizygous deletion only) and no use of LRR, approximating the settings for hapLOH.

Sensitivity is defined as the proportion of simulated aberrant markers that are called correctly. Specificity (shown in parentheses) is defined as the proportion of simulated non-aberrant markers that are called correctly. GPHMM has sensitivity less than 0.01 for purities less than 9%. Blank table entries (“-”) are due to either zero output or sensitivities <0.01. PSCN, genoCN, and ASCAT failed to produce meaningful output at all purity levels.

J-LOH maintained high sensitivity at rather low tumor purities, e.g. 0.94 at 5% tumor purity and 0.64 at 3% purity. The gain by using haplotypes can be seen via a direct comparison to results for J-LOH (*K* = 1), which had sensitivities of 0.75 and 0.24, respectively, for purities at 5% and 3%. GPHMM achieved sensitivities less than 0.01 at 5% and 3%; only at 9% did it register meaningful output. To attempt to apply all methods to the simulated data, it was necessary to simulate the LRR data, as well. Although hapLOH does not use this information (partially explaining the reduction in sensitivity relative to J-LOH), it still picked up aberrations (sensitivity 0.28) with high specificity at the 5% tumor purity. In order to gain insights on our improvement over hapLOH, we modified J-LOH to have a reduced state space of the two simulated aberration types only (hapLOH v1.0 allowed 2 aberrant states) and suppressed the use of the LRR data. This version of our method exhibited slightly lower sensitivities at 9% and 7% purities, compared with the full version that used LRR data. At lower purities, it showed greater dropoff relative to the full (LRR-informed) version of J-LOH, although it still outperformed hapLOH, most likely due to its full incorporation of the BAF data and possibly to the fact that J-LOH models the aberration states as a function of the estimated tumor proportion, which is how these data were simulated. Since hapLOH and J-LOH generate per-marker posterior probabilities for each state, we performed a full comparison among these methods via a comparison of ROC curves (Supplementary Figure S3).

**Figure 2:**
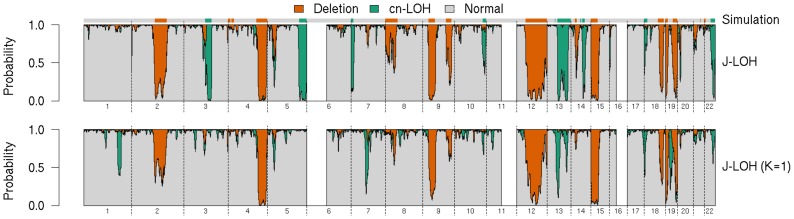
Whole genome posterior marginal probabilities for simulated 3% tumor sample. Results from J-LOH and J-LOH (*K* = 1) are presented for the simulated 3% tumor sample. The vertical bars represent the model state probabilities as in Figure 1. The horizontal bar at the top depicts the simulated aberration regions. The white gaps in the plot represent genome regions where the pure normal cell line sample shows LOH.

We compared the resolution of calls from our methods on the 3% dilution data set in Figure 2. With simulated LRR values, J-LOH is able to distinguish between deletion and cn-LOH aberrations at this low proportion. The concordance between J-LOH and J-LOH (*K* = 1) is greater for deletions than for cn-LOH for these data, since the simulated LRR deviations contribute more signal about copy number changes than does the larger perturbation in AI from cn-LOH, and both versions of the model use the LRR data equally. The haplotype-free model (*K* = 1) misses several cn-LOH events (e.g. chromosomes 3, 5, 7, and 14) and makes false calls for this aberration type (chromosomes 1, 7, 19). This illustrates that addition of the haplotype information enables discernment of aberrations particularly in the most difficult settings. We note this is a best-case scenario for these data, with the LRR values simulated from a normal distribution, offering motivation to properly model total intensity data. Below 3% tumor purity, there does not appear to be enough signal in the data to pick up regions of the size we simulated here (data not shown).

For certain settings, it may be useful to estimate the haplotype (or haplotype cluster) that is in relative imbalance. This could be used to associate the loss or gain of haplotypes with outcome, progression or some other specific phenotype [9]. In our model the over-represented allele is determined both by allele configuration and aberration state. From this we can integrate out uncertainty in the haplotypes to obtain the probability that a particular allele is over-represented (see Supplemental Information Text S2 for details). Similar to hapLOH, J-LOH has better performance than the naive method of inferring the over-represented allele by dichotomizing the BAF (Supplementary Table S1).

### Adjacent normal tissue

We also applied our method to the normal samples from paired normal-tumor samples collected for a study of hepatocellular carcinoma [10]. The adjacent normal samples were collected in order to identify and confirm aberrations in the tumor. However, here we attempted to detect aberrations that exist in the normal samples, due perhaps to contamination with tumor cells.

**Figure 3:**
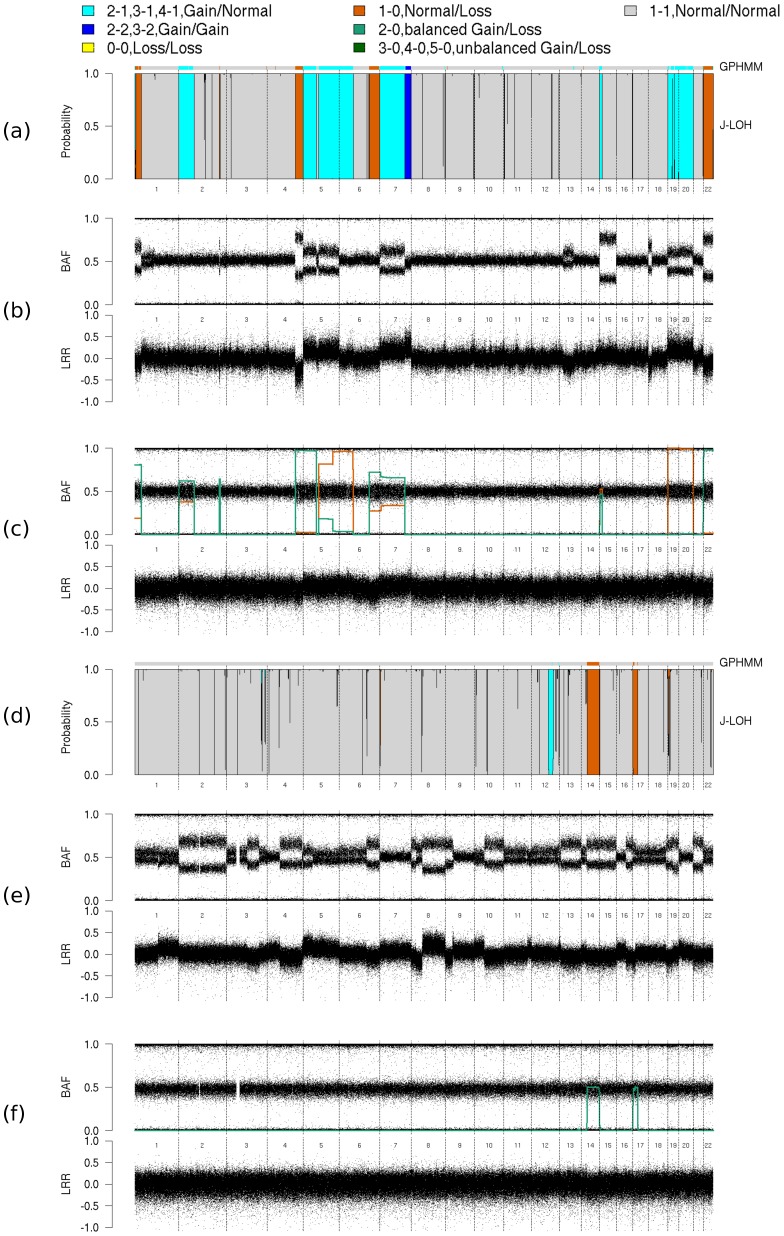
Unpaired analyses of adjacent normal samples. Posterior probabilities from the normal sample, tumor sample BAFs and LRRs, and normal sample BAFs and LRRs are presented for sample pair GSM809143/GSM809144 (a–c) and sample pair GSM809109/GSM809110 (d–f). Results from GPHMM are represented by horizontal bars above the posterior probability plots (a,d), and results from hapLOH are represented by green and orange curves (higher and lower levels of imbalance, respectively) overlaid on the BAF data (c,f).

We present results from two patients in Figure 3, along with the BAF and LRR data from the tumor samples for comparison to what we found in the adjacent normal. For the first patient (left column of Figure 3), J-LOH identifies in the normal sample some of the AI events visually evident in the tumor. While most of the AI regions identified by J-LOH are also detected with hapLOH (panel c), J-LOH appears to offer greater resolution in the specific types of events. For example, J-LOH distinguishes deletions and duplications, even though the method was applied to the BAF data only. Notably these types are consistent with LRR deviations visible in the tumor sample data (panel b). There are numerous “spikes” in the plot of the results from J-LOH, perhaps due to genotyping errors (homozygotes called as heterozygotes may exert large influence). Results from applying GPHMM were fairly consistent to those from J-LOH except on the distal portions of chromosome 1p and chromosome 22q, where GPHMM identified small regions of cn-LOH (panel a). We estimated the proportion of aberrant cells in the normal sample to be 16.2%, very close the estimate obtained from GPHMM (16.0%). However, for a second pair (right column of Figure 3), J-LOH estimated the tumor proportion in the normal sample to be 13.1%, and GPHMM estimated 12.2%. In this sample, both methods identified the cn-LOH on chromosomes 14 and 17, and the mono-allelic duplication on chromosome 12. Each of these were consistent with visual inspection of the tumor BAF/LRR. We note the regions called by GPHMM were much shorter for the events on chromosomes 12 and 17. For the chromosome 17 event, at least, the aberration that we pick up is visible in the tumor sample. Although this analysis was conducted as a small comparison among J-LOH, hapLOH and GPHMM, a quick inspection of results from the other sample pairs indicates that we often pick up large events that are not evident in the matched tumor samples (data not shown). This is possibly due to the presence of clonal mosaicism in this putatively normal tissue (e.g. [11]) or to sampling of different subclones in the tumor and the normal sample.

## Discussion

We have presented a likelihood-based framework for modeling somatic copy number changes jointly with an individual's germline haplotypes. Our method, which we term J-LOH, leverages haplotype information to differentiate subtle patterns in the BAF data from noise and also models the magnitude of observed BAF values. The main application considered here was the detection of acquired chromosomal aberrations present in tumors or other clonal populations, among DNA from mostly normal cells using SNP microarrays.

To apply our statistical approach, we made several simplifying assumptions. First, we do not model tumor heterogeneity directly but rather assume a single tumor proportion that applies to the entire genome. Second, we assume a diploid background for the tumor. As to the first assumption, our model is fairly flexible, allowing up to 21 distinct underlying states of various degrees of allelic imbalance; thus we may indirectly accommodate subclonal events via an alternate state that would exhibit a more subtle BAF deviation. Further, our model can be applied indirectly to detect subclonal aberrations by conditioning out events likely to derive from a primary clone. As to the second assumption, changes in ploidy will affect the calibration of LRR data in our model (e.g. the baseline shift). However, this should have a relatively minor impact at the lowest tumor purities that have motivated this model. Ultimately, we view J-LOH as unique in its combination of full likelihood model and haplotype information and may serve as a foundation on which further improvements may be made by relaxing certain assumptions.

Our approach offers improvements over existing methods that use either the dispersion of BAFs or their directions but fail to model both pieces of information. In data simulated from real tumor and paired normal samples run on an Illumina 370K SNP array, we can detect a cn-LOH region about 3 Mb in length at 5% purity and 10 Mb at 3% purity, analyzing unpaired samples only. At the lowest tumor purities, J-LOH showed superior performance over existing methods evaluated here. Since our model was most similar in structure to that in GPHMM, we focused most heavily on these comparisons. To attempt to isolate further the reasons for improvement offered by incorporating haplotypes, we fixed our model to a reduced state space, i.e. J-LOH (*K* = 1). The superiority of this reduced model over GPHMM at first surprised us, as we considered these models to be essentially equivalent. However, there are several key differences. GPHMM does not utilize a log R ratio scale coefficient, and is thus less flexible in fitting log R ratio data. Also, GPHMM includes an extra “fluctuation” state, which could be an advantage in certain settings but may affect the aberration calling at low tumor purities where the likelihood is essentially less peaked among states. Finally, and perhaps most importantly, GPHMM models the mBAF quantity discussed previously, whereas our model uses a mixture of normal distributions to accommodate the increased dispersion in the untransformed BAFs. Mirrored BAFs deviate severely from normality at low tumor proportions, so even the reduced version of our model which assigns equal weight to component memberships offers an additional and rapid tool for the field. The full version of the model provides further improvement over the reduced version by incorporation of germline haplotype information, which essentially provides prior weights for the component memberships.

Our method shares several attributes with another haplotype-aware method, hapLOH. Both are based on HMMs, and in this study we have applied with the same transition rates (essentially priors on aberrant event sizes and prevalence). Most obviously, both are designed to take advantage of haplotype information, with hapLOH taking phased haplotypes as input and J-LOH using a full model for population genetic information. The incorporation of a full model offers several potential advantages. First, integrating out uncertainty in haplotypes rather than using a single estimate may be useful when the distribution of haplotype estimates is rather flat (several distinct possibilities are equally likely) and this integration offers a natural mechanism for handling haplotype uncertainty. Second, J-LOH does not use consecutive 2-site haplotypes only, but rather attempts to model the phase at multiple heterozygous genotypes jointly, potentially increasing power to test larger groups of alleles together for evidence of imbalance. In addition to the distinct manner in which they incorporate haplotype information, the methods (or software packages) differ in several additional key aspects. First, whereas hapLOH makes no assumption on the distribution for BAFs at heterozygous sites in aberrant regions, J-LOH assumes normality. As a result, J-LOH may excel at sensitivity, but should be less robust with outliers and misspecified BAF distributions. Second, in its current implementation, hapLOH makes no use of log R ratio information. This diminishes its ability to differentiate among aberrant types, although *post hoc* analyses could add some of this back in a less integrated fashion. Third, our method assumes a global mixture proportion, which will be insufficiently flexible in cases where multiple clones contribute to the genomic signal, although this will be less severe when a single clone dominates the landscape. (In concurrent work, we are adapting J-LOH to accommodate heterogeneity directly.) hapLOH does not explicitly identify subclones or tumor proportion, and thus it may remain more robust to heterogeneity for inferring aberrant regions. Finally, hapLOH is fairly thrifty with computation power and allows reanalysis of imbalance without having to re-estimate the haplotypes. Therefore, it scales well with larger sample sizes.

Although we have focused here on detecting AI in low-purity tumor samples, there are numerous other applications of our method. For example, a potential problem in analyzing mixtures of tumor and normal DNA sources is the identification of germline heterozygous sites, which may be observed with error or called as missing. Our method is ideally suited for joint inference of genotypes and aberrant events, as it uses information from both the BAFs and patterns of linkage disequilibrium. This is analogous to methods for pure normal samples that attempt to infer or correct genotypes with information about population haplotypes [12], [13]. More subtle, yet important, phenomena, could also be queried with our model. One example is assessment of the relationship between specific haplotypes and copy number aberrations [9], for which our model could be applied by testing for associations with each latent haplotype cluster (a consensus or ancestral haplotype), marker-wise along the genome. Our software explicitly allows for the probabilistic identification of the cluster from which the amplified or lost chromosome derived. Essentially, this offers a haplotype-based association test, either to test the combined effect of multiple alleles on the same chromosome or to powerfully tag an untyped variant of significance.

A final possible application for J-LOH would be to take advantage of the increased sensitivity offered from careful modeling of the germline and tumor for inference of aberrations in secondary or tertiary subclones that may be at extremely low, yet variable, frequencies. Thus, due to tumor heterogeneity, methods such as ours may be important for study of the entire tumor genome even in the case when tumor purity is high. We expect there will emerge clinically relevant implications of findings from such subtle phenomena and that the incorporation of haplotype information, such as we provide here, will become an essential component in state-of-the-art software packages for analysis of somatic chromosomal aberrations using data from DNA microarrays and next-generation sequencing data.

## Materials and Methods

### The J-LOH model

Here we give a formal description of our model. We assume array data from *M* SNP markers, consisting of a B allele frequency (BAF) *b_m_*, log R ratio (LRR) *r_m_*, and genotype calls *g_m_* at each marker *m* (1,…,*M*), with *b*, *r* and 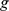 denoting the set of values at all *M* sites. For low values of tumor purity (e.g.<20%), we assume 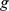 is an accurate representation of germline genotypes, since the BAF and LRR values will be minimally perturbed.

To model the observed array data, we introduce a latent variable *l_m_* to denote the aberration type in the tumor cells (e.g. deletion, cn-LOH, duplication, etc.) at marker *m* (1,…,*M*). Given the vector of specific aberration states *l* and the proportion 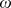 of DNA with aberrations, we assume *b* and *r* are independent, i.e.
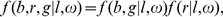
(1)an assumption made in previous approaches (e.g. in GPHMM and genoCN).

These methods have further assumed conditional independence across markers, i.e.
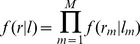
(2)and
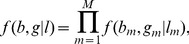
(3)greatly simplifying their computation. However, the assumption of independence in equation (3) ignores critical information provided by haplotypes, which not only summarize the dependence among SNP genotypes but also inform the expected patterns of *b* given 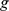 for values of *l* corresponding to AI in the tumor.

To model the dependence among observed data across markers, we leverage the expectation that chromosomal aberrations covering a contiguous region are likely due to a small number of molecular loss or gain events (often 1). We thus accommodate the dependence across markers with a model for haplotype variation. To do so, we apply a widely-used model for haplotype phasing and imputation, i.e. that underlying the software fastPHASE [14]. We start by assuming there is a set of haplotype clusters, representing ancestral haplotypes, from which haplotypes in a “present-day” sample have derived. These latent haplotype clusters, parameterized by haplotype-specific allele frequencies and relative cluster weights, capture the dependence among genotypes at different markers (or “linkage disequilibrium”), summarizing the major forms of haplotypes present in a collection of diploid samples. Specifically, assume there are *K* haplotype clusters and let *z_m_* = (*z_m_*_1_,*z_m_*_2_) indicate an ordered pair of latent clusters from which an individual's two inherited haplotypes are derived at site *m*, with 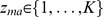, for *a* = 1,2. We observe alleles as *A* or *B* according to cluster-specific allele frequencies. Given *z_m_*_2_ = *k*, the *a^th^* allele at marker *m* is derived as a *B* with probability 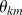, with 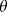 denoting the set of all such probabilities. We let 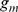 (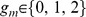, the number of inherited *B* alleles) denote the genotype at marker *m*, with the following conditional distribution:
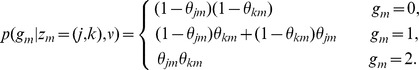



We further assume 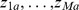 (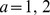) form a Markov chain on 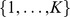, with 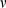 denoting all parameters for this Markov chain, including its transition probabilities and emission probabilities 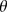. The essential difference between the model underlying fastPHASE and that used here is the ordering of cluster indicators (*z_m_*_1_,*z_m_*_2_). For clarity in our exposition, we label each component of *z_m_* as maternal or paternal (arbitrarily).

The key to our method is the integration of haplotype information and aberration event, which we accomplish by expanding the state space of *l_m_* to explicitly define an ordered pair (with respect to *z*) of states for any aberration type leading to allelic imbalance. e.g. “2-0” and “0-2” (cn-LOH), “1-0” and “0-1” (deletion), “2-1” and “1-2” (duplication), etc., plus “1-1” (normal diploid), “0-0” (homozygous deletion) and “2-2” (balanced duplication). Similar to GPHMM, we limit the maximum total copy number to 5; for our model this results in 21 states for the aberration chain (*l*).

It is worth noting that for any pair (e.g. “2-1” and “1-2”) of unbalanced aberration states, say *l*_1_ and *l*_2_, even though the full conditional (“posterior”) probability 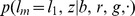 is not necessarily equal to 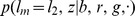, the equality is always true for 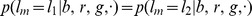, as long as we use symmetric TPMs. As a result, although there exist up to 21 internal states in the model, we report conditional probabilities for up to 12 (3 balanced states plus 9 imbalanced), after summing over the pairs.

In contrast to (3), we obtain independence across markers only by conditioning on both *l* and *z*, i.e.
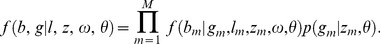
(4)


Finally, we combine equations (1), (2) and (4) and sum over possible values for *l* and *z* to obtain the following likelihood:
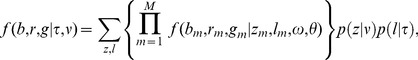
(5)where 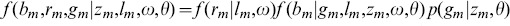 and 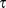 denotes the set of parameters for the conditional distributions of *b* and *r* (e.g. emissions), including, among others, the tumor proportion 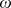.

**Figure 4:**
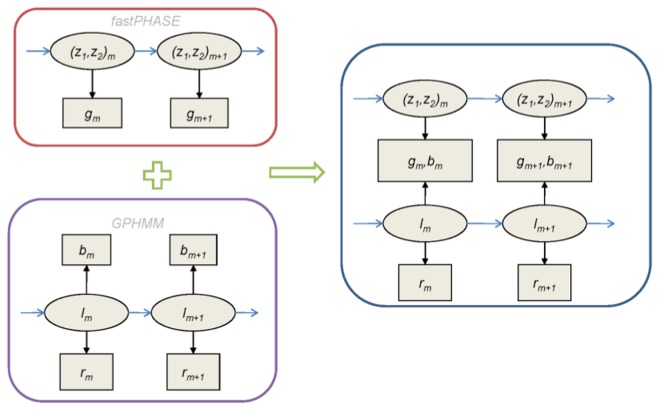
A joint model for germline haplotypes and acquired DNA aberration (J-LOH). Here we extend the HMM-based GPHMM model (bottom left) to include haplotype information, also modeled via an HMM similar to fastPHASE (top left). However, one key difference from GPHMM is that in our model, we do not use mirrored BAFs but rather model the untransformed BAF (*b*) and log R ratio (*r*) data directly. Also, unlike in fastPHASE, the pair of *z* in our model are ordered. In the joint model (right), *l*_1_,…,*l_M_* and *z*_1_,…,*z_M_* form two *a priori* independent Markov chains, with *l* describing the somatic mutation events and *z* the germline allelic dependence. The inclusion of germline genotype information contained in 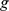 helps in better modeling dependence of observed BAF (*b_m_*) and generating more accurate posterior probabilities of aberrant states (*l_m_*).

A schematic for our joint model for haplotypes and chromosomal aberrations is depicted in Figure 4. In summary, we assume 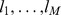 and 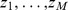 form two *a priori* independent Markov chains and *z* is ordered w.r.t. *l*, with *l* describing the somatic mutation events and *z* the allelic dependence. Estimates for 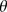 and parameters of the transition probability matrix (TPM) for *z* and can be obtained from fitting fastPHASE to external reference population data. The TPM for *l* is defined in Supplementary Text S1.

We now examine the emission functions for *b* and *r* in equation (5). Let 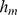 denote the ordered allele configuration, i.e. 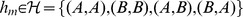. Then we can write the conditional distribution of *b* in the emission as
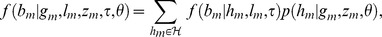
where
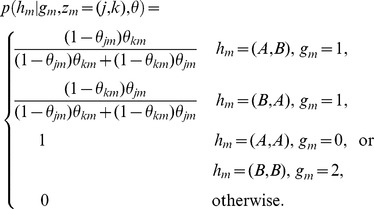



Here we have assumed the genotype calls are correct. (A more comprehensive model could allow for genotyping error by applying small deviations from the quantities above.) This assumption seems to work well for our purpose of identifying regions of aberration. In fact, in our results, after applying tQN [15] we simply called genotypes (AA, BB or AB) via simple thresholds for BAFs (e.g. 0.8 and 0.2) from mixture sample itself. Critically, at heterozygous sites (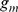  = 1) our model differentiates the two possible haplotype configurations, while methods assuming BAF independence usually equally split probability among them.

We define 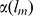 and 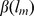 as functions on the state space of *l* that give parent-specific copy numbers. Then the distribution for the BAF at a heterozygous marker *m*, given aberration type and inherited allele configuration, is assumed to be normal with the expectation as a weighted average of the “would-be” BAF of the normal and tumor cell, that is
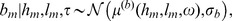
where
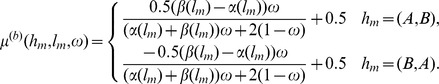



Since we ignore the possibility of somatic point mutations at these markers, at homozygous sites the BAF has a distribution that is independent of mixture proportion and aberration type; therefore, BAFs are informative for *l* at heterozygous sites only, and 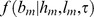 is 1 at non-heterozygous sites (either missing or homozygous). For the LRR, we assume normality, regardless of genotype, i.e.
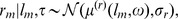
where
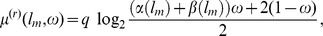
and *q* is a sample specific LRR scale coefficient.

### Parameter estimation for aberration HMM

In our model, we have assumed parameters of the population genetic model 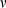 are known. In practice, we estimated these from an external reference sets of appropriate genetic ancestry; here we used the HapMap CEU panel [16]. Additionally, since the likelihood surface is multi-model for 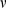, we run J-LOH with multiple estimates of 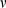, obtained from different random starting values for the EM. We then average posterior probabilities for *l* from each run of the EM for 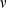, as this has worked well for other applications of this model, such as genotype imputation [14].

To estimate the tumor genome parameters for J-LOH (e.g. 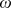, 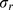, 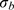, 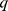 and a GC correction factor), we implemented an EM algorithm. We conducted expectation calculations (“E step”) in parallel by chromosome, as aberration events are assumed to be independent across chromosomes. Then in the “M step”, we updated global model parameters based on information from the entire genome; thus, the running time of the EM was determined by the chromosome with the greatest number of markers. The new estimates are derived via numerical methods using posterior probabilities of latent aberration states at each marker, obtained with forward and backward algorithms (see Supplementary Text S2).

Since our model accommodates a potentially large number of latent states, we have observed multiple modes of the likelihood for 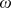. We have therefore instituted a few approaches to select starting values for 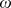 in the EM algorithm. For lab dilution samples with a range of possible tumor proportions, we first performed a grid search at proportions from 6% to 30% in steps of 2%, and then used the value that maximized the likelihood as an initial value in subsequent EM steps. For the computational dilution data, we assumed the tumor proportion was low, and selected 5% as an initial value. We generally attain convergence of our estimate for the tumor proportion 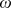 within 15 iterations.

### Software

J-LOH was written in C and compiled with openMP library for parallel computation. We compiled and ran our program a Mac Pro with two 6-core 2.66 GHZ Intel Xeon CPUs and 40 G RAM. With 319,000 SNP markers, 10 haplotype clusters and 21 aberration states, the computation time was approximately 20 seconds per EM iteration. The per iteration time was reduced to about 6 seconds with cluster count 5; and about 1 second with our haplotype-unaware model (*K* = 1). If TPM estimation is needed, the running time would increase, e.g. for cluster count 5 setting, to about 120 seconds. J-LOH and scripts for displaying output will be made available at scheet.org/software.

### Lab-based dilution samples

In this dilution series, a breast cancer cell line (ATCC CRL-2324) was mixed with the matched normal cell line (CRL-2325) at 10 different target proportions [1]. Mixed DNA samples were hybridized onto Illumina HumanCNV370 BeadChips and fluorescent signals were processed using the BeadStudio software (Illumina Inc.). We downloaded the microarray data from NCBI's GEO website (accession GSE11976). For GPHMM and J-LOH, the array data was pre-processed with the normalization tool tQN [15]; for others, we applied the genomic wave correction program from PennCNV [17] to diminish the wave effect in the log R ratio.

We applied our method along with the other methods (GPHMM 1.3, ASCAT 2.1, genoCN 1.06, PSCN 1.0, hapLOH 1.0) with default settings to SNP array data from dilution samples with 30%, 21%, 14% and 10% tumor DNA. PSCN was run with minimal segmentation size 40. GPHMM was run with a tumor proportion range of 0.02–0.5. For J-LOH, parameters for fastPHASE were estimated with a reference population using 1, 5 and 10 for the number of haplotype clusters. We ran J-LOH with 25 iterations of the EM for each random start. At each marker, the aberration state was called using the mode of the posterior probabilities.

To compare J-LOH with other methods, we first analyzed the *pure tumor sample* with GAP, which is designed for tumor samples, to come up with a “gold standard” set of calls to which we could compare results from applying various methods to the diluted tumor samples (our aim is not to compare the methods to GAP, *per se*.) GAP was chosen for this purpose for the following reasons. 1) A recent review [18] demonstrated superior performance of GAP for recall and false-discovery rate evaluation; as a conclusion the author recommended GAP for advanced users. 2) Since our main focus is to demonstrate improvement over an existing state-of-art HMM-based method (GPHMM), we utilized a break-point method to minimize bias that could result from choosing another HMM-based approach. 3) The author of GPHMM stated “the results of GAP have very good agreement with those obtained by GPHMM in the pure cancer cell line data”, so by using results from GAP for comparison we did not intentionally put GPHMM at any disadvantage.

We defined the concordance metric as the percentage of markers with calls consistent with GAP and considered the following three criteria: (a) equal total allele copy number and LOH status, which was used to define a self-consistency metric for the evaluation of GPHMM [7]; (b) gain/loss of inherited alleles, which is the natural summary of PSCN, and (c) existence of allelic imbalance, which allowed comparison with hapLOH.

We restricted the marker set to the 319,000 markers in the intersection with the HapMap CEU marker set. All markers on chromosomes 6 and 16 were excluded from further analysis, as deletions are present in the normal cell line sample [18]. For purposes of scoring concordance and other metrics, we also excluded regions based on our own GAP analyses, including LOH regions inferred to be in the normal sample and markers in GAP calls with low confidence (score  = 4) or subclonality. Additionally, since some other methods have minimum region lengths (e.g. PSCN), we also excluded short GAP calls (≤40 markers). After these actions, the denominator for calculating concordance rate consisted of 226,868 SNP markers, or about 71% of the 319,000 SNP markers originally in the intersection with HapMap.

### Low-purity computational dilution samples

We created computational dilutions with mixture proportions of tumor from 1% to 10% in steps of 1%. For selected tumor LOH regions, BAFs were interpolated from the pure normal and pure tumor BAFs, according to the intended aberration event (either deletion or cn-LOH) and mixture proportions, as described in [6]. LRR values were simulated according to the theoretical model used in GPHMM [7]. The simulated aberrations covered 72,986 markers, including 23,152 in copy-neutral LOH regions and 49,834 in hemizygous deletion regions. The lengths of the aberrant regions are between 250 and 10,720 markers, with a mean of 2,918 markers.

We ran all methods with the same settings as for the lab-based dilution samples. For J-LOH, we did not estimate the aberration state TPM with the EM as we did for lab dilutions; rather, we fixed values so that aberrant events had a mean length of 600 markers (about 20 Mb) and covered 10%. Concordance with the simulated aberrant states was calculated the same as for the lab-based dilutions but with marker exclusions as described in [6]. To calculate sensitivity and specificity for hapLOH we first grouped both aberrant states and then contrasted this total probability with that for the normal state. For other methods, including J-LOH, we used the state called by the software. In order to compare with hapLOH, we ran a modified version of our method, where the latent state space was limited to include normal, deletion and cn-LOH events only. ROC curves were obtained by choosing different thresholds for the posterior probability of being in some aberrant state.

### Adjacent normal tissue

We downloaded data from GEO (accession GSE32649) from a study of hepatocellular carcinoma [10], in which both cancer and surrounding normal tissues from 86 patients were analyzed on an Illumina 370K array. We ran J-LOH on the normal samples only, with a fixed TPM assuming that aberrant events had a mean length of 600 markers (about 20 Mb) and covered 0.1% of the genome. We visually inspected posterior probability plots and array data of matched tumor samples for aberrant regions with matching boundaries and event type.

## Supporting Information

Figure S1 **The distributions of untransformed BAF (top) and mirrored BAF (bottom) at heterozygous markers in normal regions (blue color) and allelic imbalanced regions for various magnitudes of allelic imbalance.** As the magnitude of allelic imbalance decreases, the distribution of mirrored BAF deviates more from normality.
(PDF)

Figure S2 **Schematic diagram for re-orientation process.** This diagram illustrates the re-orientation of “A/B” alleles at heterozygous markers in an allelic imbalanced chromosome region, with knowledge of haplotypes information. In an allelic imbalanced region, the BAF has either a “shifted up” (blue) or “shifted down” (red) distribution, forming two bands on opposite sides of 0.5. We reverse “A/B” allele as necessary such that one chromosome carries all “A” alleles at heterozygous markers while the other all “B” alleles. Accordingly, the observed BAF at a marker of which the allele label is changed would be replaced with its complement (1-BAF). For example, if the original BAF has the red distribution, the complement (1-BAF) would have the blue distribution. The BAFs after re-orientation become “one-band” and maintain normality. In contrast, the distribution of the mirrored BAF is bounded by 0.5 and distorted.
(PDF)

Figure S3 **Comparison of ROCs between hapLOH and J-LOH at tumor purities 3% and 5%**. We first classify existence of any aberration state by applying different thresholds to the posterior probability of being normal to obtain the ROC. Since hapLOH uses only the BAF information, we ran J-LOH first with both BAF and LRR and then with BAF inputs only.
(PDF)

Table S1 **Accuracy of various methods for inferring over-represented alleles.** At a heterozygous marker in allelic imbalanced regions, either allele “A” or “B” is over-represented in the tumor. We compare the accuracy for inferring over-represented alleles in the tumor. Accuracy is defined as the proportion of heterozygous markers in AI regions where the over-represented alleles are inferred correctly. The true over-represented alleles are ascertained from dichotomizing BAFs in AI regions in pure tumor cell line. The naive method selects the allele by comparing the mixture BAF to 0.5. Both J-LOH and hapLOH infer with enhanced accuracy.
(PDF)

Text S1 **Transitions probability matrix for the aberration state HMM.**
(PDF)

Text S2 **Estimation of model parameters.**
(PDF)
