# Supplementary material for: Identification of Allelic Imbalance with a Statistical Model for Subtle Genomic Mosaicism
Source: PLoS Comput Biol. 2014 Aug 28;10(8):e1003765. doi: 10.1371/journal.pcbi.1003765 (PMC4148184; doi:10.1371/journal.pcbi.1003765)
Supplement: Figure S1 — The distributions of untransformed BAF (top) and mirrored BAF (bottom) at heterozygous markers in normal regions (blue color) and allelic imbalanced regions for various magnitudes of allelic imbalance. As the magnitude of allelic imbalance decreases, the distribution of mirrored BAF deviates more from normality. (PDF) [file pcbi.1003765.s001.pdf]

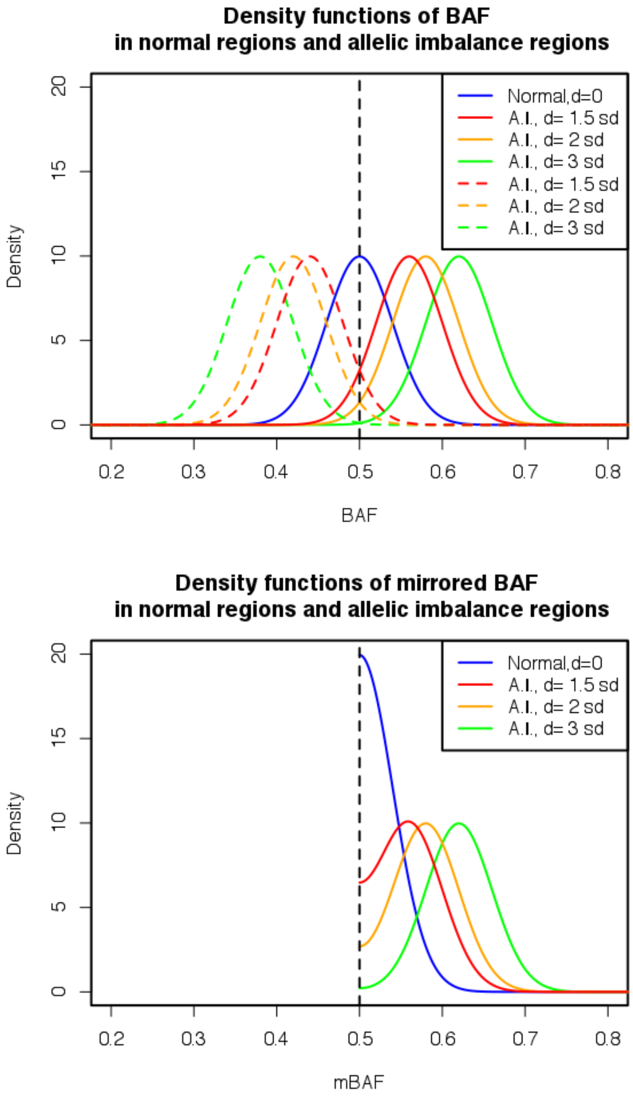


Figure S1: **The distributions of untransformed BAF (top) and mirrored BAF (bottom) at heterozygous markers in normal regions (blue color) and allelic imbalanced regions for various magnitudes of allelic imbalance.** As the magnitude of allelic imbalance decreases, the distribution of mirrored BAF deviates more from normality. Compared to using the untransformed BAF, the critical value of a one-side test using the mBAF is greater; the area under the alternative density (power) is smaller. Compared to that of untransformed BAF, the ratio of AI state density of mBAF to normal state density of mBAF, at a typical value for AI state, is smaller; therefore the hidden Markov model posterior probability for being in an AI state, at markers in AI regions, is smaller.
