# Supplementary material for: Identification of Allelic Imbalance with a Statistical Model for Subtle Genomic Mosaicism
Source: PLoS Comput Biol. 2014 Aug 28;10(8):e1003765. doi: 10.1371/journal.pcbi.1003765 (PMC4148184; doi:10.1371/journal.pcbi.1003765)
Supplement: Figure S2 — Schematic diagram for re-orientation process. This diagram illustrates the re-orientation of “A/B” alleles at heterozygous markers in an allelic imbalanced chromosome region, with knowledge of haplotypes information. In an allelic imbalanced region, the BAF has either a “shifted up” (blue) or “shifted down” (red) distribution, forming two bands on opposite sides of 0.5. We reverse “A/B” allele as necessary such that one chromosome carries all “A” alleles at heterozygous markers while the other all “B” alleles. Accordingly, the observed BAF at a marker of which the allele label is changed would be replaced with its complement (1-BAF). For example, if the original BAF has the red distribution, the complement (1-BAF) would have the blue distribution. The BAFs after re-orientation become “one-band” and maintain normality. In contrast, the distribution of the mirrored BAF is bounded by 0.5 and distorted. (PDF) [file pcbi.1003765.s002.pdf]

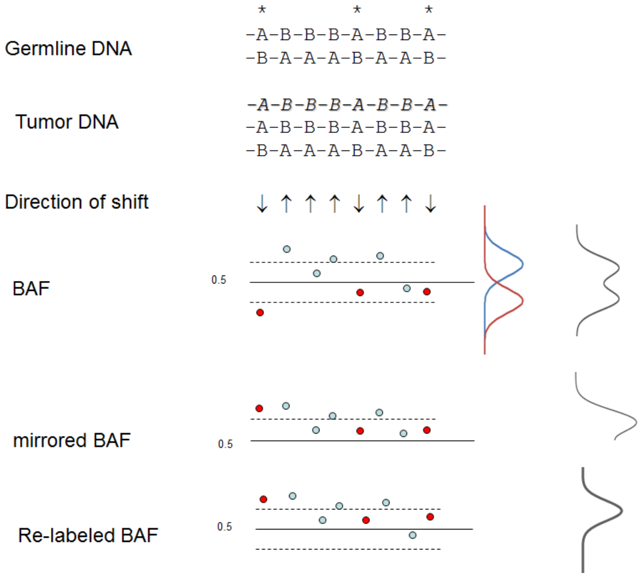

Figure S2: **Schematic diagram for re-orientation process.** This diagram illustrates the re-orientation of "A/B" alleles at heterozygous markers in an allelic imbalanced chromosome region, with knowledge of haplotypes information. In an allelic imbalanced region, the BAF has either a "shifted up" (blue) or "shifted down" (red) distribution, forming two bands on opposite sides of 0.5. We reverse "A/B" allele as necessary such that one chromosome carries all "A" alleles at heterozygous markers while the other all "B" alleles. Accordingly, the observed BAF at a marker of which the allele label is changed would be replaced with its complement (1-BAF). For example, if the original BAF has the red distribution, the complement (1-BAF) would have the blue distribution. The BAFs after re-orientation become "one-band" and maintain normality. In contrast, the distribution of the mirrored BAF is bounded by 0.5 and distorted.
