# Supplementary material for: Identification of Allelic Imbalance with a Statistical Model for Subtle Genomic Mosaicism
Source: PLoS Comput Biol. 2014 Aug 28;10(8):e1003765. doi: 10.1371/journal.pcbi.1003765 (PMC4148184; doi:10.1371/journal.pcbi.1003765)
Supplement: Figure S3 — Comparison of ROCs between hapLOH and J-LOH at tumor purities 3% and 5%. We first classify existence of any aberration state by applying different thresholds to the posterior probability of being normal to obtain the ROC. Since hapLOH uses only the BAF information, we ran J-LOH first with both BAF and LRR and then with BAF inputs only. (PDF) [file pcbi.1003765.s003.pdf]

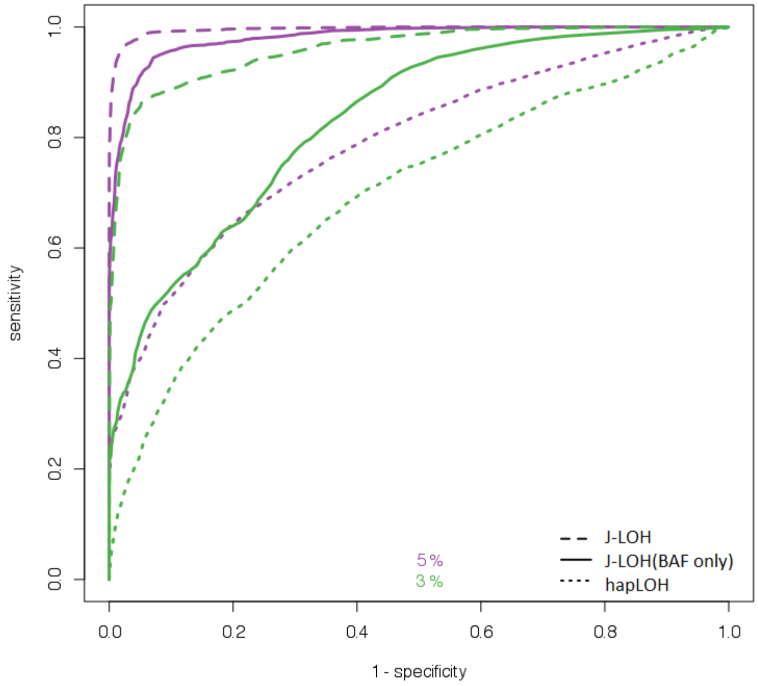

Figure S3: **Comparison of ROCs between hapLOH and J-LOH at tumor purities 3% and 5%.** We first classify existence of any aberration state by applying different thresholds to the posterior probability of being normal to obtain the ROC. Since hapLOH uses only the BAF information, we ran J-LOH first with both BAF and LRR and then with BAF inputs only.
