# Supplementary material for: Identification of Allelic Imbalance with a Statistical Model for Subtle Genomic Mosaicism
Source: PLoS Comput Biol. 2014 Aug 28;10(8):e1003765. doi: 10.1371/journal.pcbi.1003765 (PMC4148184; doi:10.1371/journal.pcbi.1003765)
Supplement: Table S1 — Accuracy of various methods for inferring over-represented alleles. At a heterozygous marker in allelic imbalanced regions, either allele “A” or “B” is over-represented in the tumor. We compare the accuracy for inferring over-represented alleles in the tumor. Accuracy is defined as the proportion of heterozygous markers in AI regions where the over-represented alleles are inferred correctly. The true over-represented alleles are ascertained from dichotomizing BAFs in AI regions in pure tumor cell line. The naive method selects the allele by comparing the mixture BAF to 0.5. Both J-LOH and hapLOH infer with enhanced accuracy. (PDF) [file pcbi.1003765.s004.pdf]

	computational dilut.				lab dilut.	
	3%	5%	7%	9%	10%	21%
Naive	0.60	0.67	0.73	0.78	0.85	0.97
J-LOH	0.76	0.86	0.92	0.94	0.95	0.99
hapLOH	0.75	0.84	0.90	0.93	-	-

Table S1: **Accuracy of various methods for inferring over-represented alleles.** At a heterozygous marker in allelic imbalanced regions, either allele "A" or "B" is over-represented in the tumor. We compare the accuracy for inferring over-represented alleles in the tumor. Accuracy is defined as the proportion of heterozygous markers in AI regions where the over-represented alleles are inferred correctly. The true over-represented alleles are ascertained from dichotomizing BAFs in AI regions in pure tumor cell line. The naive method selects the allele by comparing the mixture BAF to 0.5. Both J-LOH and hapLOH infer with enhanced accuracy.
