# Supplementary material for: Identification of Allelic Imbalance with a Statistical Model for Subtle Genomic Mosaicism
Source: PLoS Comput Biol. 2014 Aug 28;10(8):e1003765. doi: 10.1371/journal.pcbi.1003765 (PMC4148184; doi:10.1371/journal.pcbi.1003765)
Supplement: Text S1 — Transitions probability matrix for the aberration state HMM. (PDF) [file pcbi.1003765.s005.pdf]

Transitions probability matrix for the aberration state HMM

Consistent with the model in hapLOH [1], all aberrant states are equal *a priori*, while different from the non-aberrant (normal) state. This is in contrast to GPHMM [2], which treats the normal and aberrant states equally. We use 3 parameters to describe the transition probability matrix (TPM). Let p_0 be the probability that next state is any aberrant state given that the current state is the normal, p_1 be the probability that next state is a different state (normal or another aberrant state) given that the current state is an aberrant state, and p_2 be the probability that next state is another aberrant state, given that the current is aberrant state and a transition occurs. The probabilities (p_0, p_1, p_2) then induce the following TPM:

$$\begin{array}{c} \\ Normal \\ Aberr_1 \\ \vdots \\ Aberr_N \end{array} \begin{array}{c} \begin{array}{cccc} Normal & Aberr_1 & \dots & Aberr_N \end{array} \\ \begin{pmatrix} 1-p_0 & \frac{p_0}{N} & \cdots & \frac{p_0}{N} \\ p_1(1-p_2) & 1-p_1 & \cdots & \frac{p_1p_2}{(N-1)} \\ \vdots & \vdots & \ddots & \vdots \\ p_1(1-p_2) & \frac{p_1p_2}{(N-1)} & \cdots & 1-p_1 \end{pmatrix} \end{array},$$

where N (up to 20 in our implementation; see main text) is the number of aberrant states. The three parameters (p_0, p_1, p_2) can be optionally estimated by keeping track of the probabilities $p^{(i)}(l_m, l_{m+1}|g, b, r)$ at the ith iteration in the usual way. We also use (p_0, p_1, p_2) to compute the stationary distributions for the starts of HMMs.

References

[1] Selina Vattathil and Paul Scheet. Haplotype-based profiling of subtle allelic imbalance with snp arrays. *Genome Research*, 23(1):152–158, 2013.

[2] A. Li, Z. Liu, K. Lezon-Geyda, S. Sarkar, D. Lannin, V. Schulz, I. Krop, E. Winer, L. Harris, and D. Tuck. GPHMM: an integrated hidden markov model for identification of copy number alteration and loss of heterozygosity in complex tumor samples using whole genome snp arrays. *Nucleic Acids Research*, 39(12):4928–4941, 2011.
