# Supplementary material for: Identification of Allelic Imbalance with a Statistical Model for Subtle Genomic Mosaicism
Source: PLoS Comput Biol. 2014 Aug 28;10(8):e1003765. doi: 10.1371/journal.pcbi.1003765 (PMC4148184; doi:10.1371/journal.pcbi.1003765)
Supplement: Text S2 — Estimation of model parameters. (PDF) [file pcbi.1003765.s006.pdf]

Expected complete data log-likelihood and EM

In our EM algorithm, the expected complete data log-likelihood ("Q") is a function of a set of model parameters τ, i.e.

$$Q(\tau) = \sum_{m=1}^{M} \left(\sum_{z_m, l_m} log\left(f(b_m, r_m, g_m | z_m, l_m, \tau)\right) p_m^*(z_m, l_m) \right),$$

where M is the total marker number, m is the SNP marker index, b_m is the observed BAF, r_m is the observed LRR, g_m is the error-free genotype, $z_m = (z_{m1}, z_{m2})$ is ordered haplotype cluster memberships, l_m is the aberration type, τ is the model parameters set, $p_m^*(z_m, l_m) \equiv p(z_m, l_m | \tau^*, b, r, g)$ is the conditional marginal distribution, given parameter estimates τ^*. We further assume that conditioned on (z_m, l_m), r_m and (g_m, b_m) are independent (see Materials and Methods). Thus

$$Q(\tau) = \sum_{m=1}^{M} \left(\sum_{z_m, l_m} \left(log\left(f(r_m | l_m, \tau)\right) + log\left(f(b_m, g_m | z_m, l_m, \tau)\right)\right) p_m^*(z_m, l_m) \right).$$

We maximize Q at each EM cycle by solving the equation that sets to zero its partial derivative w.r.t. each parameter. For some parameters, a closed-form solution is available; for others, a numerical method must be applied.

In our experience, when the tumor mixture is high (e.g. above 10%), we can approximate the M-step by maximizing Q w.r.t. each individual parameter in τ marginally, rather than maximizing in a multivariate manner. However, for extreme low tumor purity (e.g. about 3%), to avoid convergence problems, we must take the approach of expected conditional maximization (ECM), meaning we have to re-compute the posterior probability of latent states with the updated estimates after maximizing each parameter. The computation is more expensive with ECM.

Estimation of the mixture proportion

The derivative of Q w.r.t. tumor DNA mixture proportion (w) is composed of the following two summations involving derivatives of BAF and LRR densities respectively:

$$\frac{\partial}{\partial w} Q(w) = \sum_{m=1}^{M} \left(\sum_{z_m, l_m} \left(\frac{\partial}{\partial w} log(f(r_m | l_m, \tau)) \right) p_m^*(z_m, l_m) \right) +$$
$$\sum_{m \in \{i\, st\, g_i = 1\}} \left(\sum_{z_m, l_m} \left(\frac{\partial}{\partial w} log(f(b_m, g_m | z_m, l_m, \tau)) \right) p_m^*(z_m, l_m) \right), \quad (1)$$

where M is the total number of SNP makers, and the inner sum is over all combinations of z and l. Since BAFs are informative at heterozygous sites only (germline homozygous sites

have the derivative of zero w.r.t. w), the second summation in equation (1) is limited to germline heterozygous sites.

We assume LRRs follow the same normal distribution as defined in GPHMM except for the addition of a sample-specific scale factor, i.e.

$$f(r|l,w,o_r,\sigma_r^2,q) = \frac{1}{\sigma_r}\phi\left(\frac{r-\mu^{(r)}(l,w,q)-o_r}{\sigma_r}\right),$$

where

$$\mu^{(r)}(l,w,q) \equiv q\cdot\log_2\frac{(1-w)2+w(\alpha(l)+\beta(l))}{2}, \text{ and}$$

ϕ is pdf of the standard normal distribution, l the latent aberration type, σ_r^2 the variance, o_r the global baseline shift, and q the LRR scale. The functions $\alpha(l_m)$ and $\beta(l_m)$ have domains on the state space of l and give parent-specific allele copy numbers. The derivative in the first summation of equation [1] is

$$\frac{\partial}{\partial w}log(f(r_m|l_m,\tau)) =$$
$$\frac{(r_m-o_r-q\log_2\frac{(1-w)2+w(\alpha(l_m)+\beta(l_m))}{2})}{\sigma_r^2}\cdot\frac{q(-1+0.5(\alpha(l_m)+\beta(l_m)))}{log_e(2)}.$$

We focus on low purity samples, where the perturbed BAF will remain relatively close to one-half and the truncation of BAFs at 0 or 1 (for heterozygotes) is of minimal concern. Thus, at germline heterozygous sites, we assume the potentially mixed BAF is distributed as

$$f(b|h,l,w,o_b,\sigma_b^2) = \frac{1}{\sigma_b}\phi\left(\frac{b-\mu^{(b)}(h,l,w)-o_b}{\sigma_b}\right),$$

where ϕ is the pdf of the standard normal distribution, σ_b^2 is the variance of BAF, o_b is a global baseline shift, h is the inherited allele configuration (either “AB" or “BA") and

$$\mu^{(b)}(h,l,w) \equiv \frac{0.5w\left(\beta(l)-\alpha(l)\right)(-1)^{\mathbb{1}(h=\text{“}AB\text{”})}}{(1-w)2+w\left(\alpha(l)+\beta(l)\right)}+0.5.$$

For simplicity, we subtract 0.5 from observed BAFs, then we can drop 0.5 from $\mu^{(b)}(h,l,w)$ expression and it has opposite signs for allele configurations “AB" and “BA". The derivative in the second summation of equation (1) is

$$\frac{\partial}{\partial w}logf(b_m,g_m=1|z_m=(j,k),l_m,w) = \frac{1}{\sigma_b^2}\left((b_m-o_b)\frac{1-\Omega_m}{1+\Omega_m}-\mu_m^{AB}\right)\frac{\partial}{\partial w}\mu_m^{AB},$$

where

$$\Omega_m \equiv exp\left(\frac{-2b_m\mu_m^{AB}}{\sigma_b^2}\right)\frac{p(h_m = \text{“}BA\text{”}|z_m = (j,k)}{p(h_m = \text{“}AB\text{”}|z_m = (j,k))} = exp\left(\frac{-2b_m\mu_m^{AB}}{\sigma_b^2}\right)\frac{\theta_{jm}(1-\theta_{km})}{\theta_{km}(1-\theta_{jm})},$$

$$\mu_m^{AB} \equiv \mu^{(b)}(h_m = \text{“}AB\text{”}, l_m, w) = \frac{-0.5\,(\alpha(l_m)-\beta(l_m))\,w}{(1-w)2+(\alpha(l_m)+\beta(l_m))\,w},$$

$$\frac{\partial}{\partial w}\mu_m^{AB} = \frac{-\alpha(l_m)+\beta(l_m)}{((\alpha(l_m)+\beta(l_m)-2)\,w+2)^2}, \text{ and}$$

θ_{im} is the probability that allele is “B” given haplotype cluster membership is i at maker m, as defined in fastPHASE model [1].

After substituting the two derivatives in equation (1), we do not have a closed-form solution. Therefore we rely on numerical root-finding methods. In practice, we use the secant method with previous w estimates as initial values.

Estimation of BAF global baseline shift (o_b)

The derivative of Q w.r.t. o_b is

$$\frac{\partial}{\partial o_b}Q(o_b) = \sum_{m\in\{i\,st\,g_i=1\}}\left(\sum_{z_m,l_m}\frac{\partial}{\partial o_b}log(f(b_m,g_m|z_m,l_m,\tau))p_m^*(z_m,l_m)\right).$$

Therefore, the new estimate of o_b is

$$\hat{o_b} = \frac{1}{M^{het}}\sum_{m\in\{i\,st\,g_i=1\}}\sum_{z_m,l_m}\left(b_m-\mu_m^{AB}\frac{1-\Omega_m}{1+\Omega_m}\right)p_m^*(z_m,l_m),$$

where M^{het} is the number of germline heterozygous SNP markers.

Estimation of BAF variance (σ_b^2)

The derivative of Q w.r.t. σ_b^2 is

$$\frac{\partial}{\partial \sigma_b^2}Q(\sigma_b^2) = \sum_{m\in\{i\,st\,g_i=1\}}\left(\sum_{z_m,l_m}\frac{\partial}{\partial \sigma_b^2}log(f(b_m,g_m|z_m,l_m,\tau))p_m^*(z_m,l_m)\right).$$

And using the normality assumption for BAF distribution,

$$\frac{\partial}{\partial \sigma_b^2}log(f(b_m,g_m=1|z_m=(j,k),l_m,w)) =$$
$$\frac{1}{2\sigma_b^4}\left(-\sigma_b^2+(b_m-o_b)^2+(\mu_m^{AB})^2-2(b_m-o_b)(\mu_m^{AB})\frac{1-\Omega_m}{1+\Omega_m}\right).$$

We apply numerical root-finding method to obtain the new estimate.

Estimation of variance and global baseline shift for LRR (σ_r^2, o_r)

It is easy to show that the solutions that maximize Q w.r.t. σ_r^2 and o_r are the following expressions:

$$\hat{o_r} = \frac{1}{M}\sum_{m=1}^{M}\sum_{l_m}\left(r_m - \mu^{(r)}(l_m, w)\right)p_m^*(l_m)$$

and

$$\hat{\sigma_r^2} = \frac{1}{M}\sum_{m=1}^{M}\sum_{l_m}\left(r_m - \mu^{(r)}(l_m, w) - o_r\right)^2 p_m^*(l_m),$$

where $p_m^*(l_m) = \sum_{z_m} p_m^*(z_m, l_m)$.

Estimation of LRR scale coefficient (q)

It has been pointed out that amplitude of LRR varies from sample to sample and that the observed amplitude is usually smaller than the standard value $log_2(\frac{\text{tumor copy number}}{2})$ [2]. In GAP, this is modeled with a simple coefficient of contraction that is specific to the sample. GPHMM models the expected LRR as

$$\mu^{(r)}(l, w) \equiv 2log_{10}(2) \cdot log_2\left(\frac{\text{averge allele copy number in mixture}}{2}\right).$$

In our model, extra flexibility is achieved by replacing the constant $2log_{10}(2)$ in GPHMM with a LRR scale parameter (q) and the new estimate for updating q is

$$\hat{q} = \frac{\sum_{m=1}^{M}\sum_{z_m,l_m} p_m^*(z_m, l_m)(r_m - o_r)\log_2\frac{(1-w)2+w(\alpha(l_m)+\beta(l_m))}{2}}{\sum_{m=1}^{M}\sum_{z_m,l_m} p_m^*(z_m, l_m)\left(\log_2\frac{(1-w)2+w(\alpha(l_m)+\beta(l_m))}{2}\right)^2}.$$

Estimation of a GC content coefficient

Local GC content may induce a “wave” effect in the LRR data [3]. Therefore adjusting for GC content can reduce the noise in LRR signal, as demonstrated in GPHMM [4]. Similar to GPHMM, we use average GC-percentage in a 1Mb window around each SNP maker.

Let x_m, ($m = 1 \cdots M$) denote the average GC content at marker m and t a global coefficient for GC content. Then we can re-write the density for LRR data as

$$f(r_m|x_m, l_m, w, o_r, \sigma_r^2, q, t) = \frac{1}{\sigma_r}\phi\left(\frac{r - \mu^{(r)}(l_m, w, q) - o_r - t \cdot x_m}{\sigma_r}\right).$$

It is easy to show the estimate for t is

$$\hat{t} = \frac{\sum_{m=1}^{M} \sum_{z_m, l_m} p_m^*(z_m, l_m)(r_m - o_r - \mu^{(r)}(l_m, w, q))x_m}{\sum_{m=1}^{M} \sum_{z_m, l_m} p_m^*(z_m, l_m) x_m^2}.$$

The above estimations for rest of the parameters remain valid if we replace r_m with $r_m - t \cdot x_m$.

Identification of over-represented allele in tumor DNA

After the EM algorithm converges, the latent aberration state and haplotype cluster membership at marker m has joint posterior probability $p^c(z_m, l_m) = p(z_m, l_m | g, r, b, \nu, \hat{\tau})$. We then compute the probability that the allele "B" is over-represented at a germline heterozygous marker m as follows:

$$\sum_{z_m, l_m} p(\text{“B” is over-represented} | z_m, l_m) p_m^c(z_m, l_m) =$$
$$\sum_{z_m, l_m} \sum_{h_m \in \{(\text{A,B}) \text{ , } (\text{B,A})\}} \mathbb{1}\{\text{“B” is over-presented} | h_m, l_m\} p(h_m | z_m) p_m^c(z_m, l_m),$$

where $\mathbb{1}\{\cdot\}$ is an indicator function. The probability for the allele "A" can be similarly obtained.

Mean copy of haplotype cluster in tumor DNA

It is possible that a causal factor is correlated with a particular haplotype background, either due to an untyped "causal" germline allele well tagged by a haplotype or to a "haplotype effect" itself. Therefore it may be helpful to test the association of phenotypes with the mean copy number of a haplotype cluster. Suppose we obtain the posterior probability $p_m^c(z_m, l_m)$ as defined above, the mean copy of haplotype cluster k at marker m is

$$\sum_{z_m, l_m} \left(\mathbb{1}\{z_{m1} = k\} \alpha(l_m) + \mathbb{1}\{z_{m2} = k\} \beta(l_m) \right) p_m^c(z_m, l_m),$$

where $z_m = (z_{m1}, z_{m2})$.

References

[1] P. Scheet and M. Stephens. A fast and flexible statistical model for large-scale population genotype data: applications to inferring missing genotypes and haplotypic phase. *The American Journal of Human Genetics*, 78(4):629–644, 2006.

[2] T. Popova, E. Manié, D. Stoppa-Lyonnet, G. Rigaill, E. Barillot, M.H. Stern, et al. Genome Alteration Print (GAP): a tool to visualize and mine complex cancer genomic profiles obtained by SNP arrays. *Genome Biology*, 10(11):R128, 2009.

[3] Sharon J Diskin, Mingyao Li, Cuiping Hou, Shuzhang Yang, Joseph Glessner, Hakon Hakonarson, Maja Bucan, John M Maris, and Kai Wang. Adjustment of genomic waves in signal intensities from whole-genome SNP genotyping platforms. *Nucleic Acids Research*, 36(19):e126–e126, 2008.

[4] A. Li, Z. Liu, K. Lezon-Geyda, S. Sarkar, D. Lannin, V. Schulz, I. Krop, E. Winer, L. Harris, and D. Tuck. GPHMM: an integrated hidden markov model for identification of copy number alteration and loss of heterozygosity in complex tumor samples using whole genome snp arrays. *Nucleic Acids Research*, 39(12):4928–4941, 2011.
